# Drug Synergism of Anticancer Action in Combination with Favipiravir and Paclitaxel on Neuroblastoma Cells

**DOI:** 10.3390/medicina60010082

**Published:** 2023-12-30

**Authors:** Hasan Turkez, Mehmet Enes Arslan, Harun Selvitopi, Abdurrahim Kadi, Sena Oner, Adil Mardinoglu

**Affiliations:** 1 Department of Medical Biology, Faculty of Medicine, Atatürk University, 25240 Erzurum, Turkey; 2 Department of Molecular Biology and Genetics, Faculty of Science, Erzurum Technical University, 25050 Erzurum, Turkey; enes.aslan@erzurum.edu.tr (M.E.A.); abdurrahimkadi@gmail.com (A.K.); senaoner02@gmail.com (S.O.); 3 Department of Mathematics, Faculty of Science, Erzurum Technical University, 25050 Erzurum, Turkey; harun.selvitopi@erzurum.edu.tr; 4 Science for Life Laboratory, KTH-Royal Institute of Technology, SE-17121 Stockholm, Sweden; 5 Centre for Host-Microbiome Interactions, Faculty of Dentistry, Oral & Craniofacial Sciences, King’s College London, London SE1 9RT, UK

**Keywords:** paclitaxel, favipiravir, synergistic effect, anticancer, genotoxicity

## Abstract

*Background and Objectives*: Favipiravir (FPV) is an antiviral medication and has an inhibitory effect on Cytochrome P450 (CYP2C8) protein, which is mainly involved in drug metabolism in the liver, and the expression of this gene is known to be enhanced in neuronal cells. The metabolization of Paclitaxel (PTX), a chemotherapeutic drug used in cancer patients, was analyzed for the first time in the human SH-SY5Y neuroblastoma cell line for monitoring possible synergistic effects when administered with FPV. *Materials and Methods*: Further, in vitro cytotoxic and genotoxic evaluations of FPV and PTX were also performed using wide concentration ranges in a human fibroblast cell culture (HDFa). Nuclear abnormalities were examined under a fluorescent microscope using the Hoechst 33258 fluorescent staining technique. In addition, the synergistic effects of these two drugs on cultured SH-SY5Y cells were determined by MTT cell viability assay. In addition, the death mechanisms that can occur in SHSY-5Y were revealed by using the flow cytometry technique. *Results*: Cell viability analyses on the HDFa healthy cell culture showed that both FPV and PTX have inhibitory effects at higher concentrations. On the other hand, there were no significant differences in nuclear abnormality numbers when both of the compounds were applied together. Cell viability analyses showed that FPV and PTX applications have higher cytotoxicity, which indicated synergistic toxicity against the SHSY-5Y cell line. Also, PTX exhibited higher anticancer properties against the neuroblastoma cell line when applied with FPV, as shown in both cytotoxicity and flow cytometry analyses. *Conclusions*: In light of our findings, the anticancer properties of PTX can be enhanced when the drug application is coupled with FPV exposure. Moreover, these results put forth that the anticancer drug dosage should be evaluated carefully in cancer patients who take COVID-19 treatment with FPV.

## 1. Introduction

In December 2019, a new coronavirus disease emerged in Wuhan, the capital of China’s Hubei Province [1]. This emerging new coronavirus disease is called COVID-19. WHO has declared the COVID-19 pandemic a public health emergency of international concern. Epidemiological studies show that COVID-19 is transmitted by respiratory droplets from symptomatic persons, direct contact with infected persons or persons in contact with objects and surfaces. Based on data from clinical and virological studies collecting biological samples from diagnosed patients, the type of pneumonia caused by COVID-19 disease is considered to be highly contagious. It shows that the COVID-19 virus is highest in the upper respiratory (nose and throat) tracts at the beginning of the disease course [2,3,4]. As of 27 December 2020, a total of 81,113,925 confirmed cases of 2019-nCoV and 1,772,121 deaths were reported in 224 countries [5]. Favipiravir (FPV) is one of the drugs that could potentially be used in the fight against COVID-19. This drug is a pyrazine carboxamide derivative with antiviral activity against various RNA viruses (including influenza virus, rhinovirus and respiratory syncytial virus). This drug was later approved in Japan for use in the treatment of flu [6]. Although favipiravir was originally developed against influenza virus infections, it is currently used therapeutically against a wide variety of negative-strand and positive-strand RNA viruses for which there are no licensed treatment options [7].

Paclitaxel (PTX) is a chemotherapeutic drug that is used clinically in cancer treatments such as breast, lung, ovarian, cervical and pancreatic cancers. PTX is a broadly used chemotherapeutic agent for cancer treatments, which can cause peripheral neuropathy. PTX interacts with the inner side of the microtubules and fixes the microtubule, limiting its depolymerization. When cancer cell division occurs, PTX interrupts normal spindle machinery, inhibits mitosis and finally leads to cell death [8,9]. Also, non-dividing neuronal cells are responsive to PTX treatment, and the mechanisms related to PTX neurotoxicity are not fully known yet [10]. The discovery of treatments for PTX-induced peripheral neuropathy may be precluded by identifying the mechanisms by which PTX damages peripheral axons [11]. In vitro studies showed that PTX treatment is effective against gliomas and brain metastases. On the other hand, PTX exhibited only moderate activity in patients with recurrent primary brain tumors. The ineffectiveness of drug administration is associated with a targeted failure of PTX to reach the brain, which is thought to be due to the high activity of the multidrug-resistant efflux pump P-glycoprotein (P-gp) in the apical membrane of brain endothelial cells [12].

Cytochrome P450 CYP2C8 is one of the most abundant CYP2C enzymes in the human liver after CYP2C9, and the enzyme plays an important role in the metabolism of drugs in the body. It was shown that Cytochrome P450 can metabolize xenobiotics in brain tissues [13,14]. It metabolizes antidiabetic, anticancer, cholesterol-lowering, antiarrhythmic and antimalarial drugs. The CYP2C8 gene is found at high rates in the human liver and in extrahepatic tissues such as the lung, kidney, arteries, nasal mucosa, endothelial mucosa and heart. Induction of CYP2C8 by xenobiotics may lead to a change in drugs, as it contributes to the variability in drug metabolism in humans. This change may result in therapeutic failure or drug tolerance [15]. CYP2C8 is primarily involved in the drug metabolism of amiodarone, amodiaquine (AQ), montelukast, cerivastatin (CER), repaglinide, PTX, troglitazone and rosiglitazone (RG) [16]. Moreover, most of the drug formulations utilized for COVID-19 treatment are metabolized by cytochrome P450 enzymes. This is further complicated by the genetic polymorphisms of ACE, ACE-2, HO-1 and CYP2D6, and current therapy can overcome this problem by focusing on the high activities of oxygenase enzymes [17].

For this purpose, it was thought that FPV may increase the effectiveness of the anticancer agents used by playing a role in the inhibition of Cytochrome P450 (CYP2C8), which is primarily involved in anticancer drug metabolism. For this reason, the potential of using FPV, which is widely used in the treatment of coronavirus infection with antitumor drugs in cancer patients, causing a synergistic effect and leading to a toxicological outcome, was investigated. In this way, it may be recommended to change the drug dose rates in cancer patients or to limit the use of FPV in cancer patients.

## 2. Materials and Methods

### 2.1. Cell Cultures

The human dermal fibroblast cell (HDFa, ATCC^®^ PCS-201-012™) culture was grown in the DMEM medium supplemented with 1% penicillin/streptomycin, 10% FBS and 5% CO_2_ at a 37 °C temperature until the cultures reached 80% confluency. The human neuroblastoma cell (SH-SY5Y, ATCC^®^ CRL-2266™) line was cultured in DMEM/F12 culture media containing 1% penicillin/streptomycin and 10% fetal bovine serum at 37 °C and 5% CO_2_. Neuroblastoma cell cultures were incubated to grow up to 80% cell density.

### 2.2. Cytotoxicity and Cell Viability Analyses

Cell viability analyses were performed on HDFa and SH-SY5Y by using the 3-(4,5-dimethylthiazol-2-yl)-2,5-diphenyltetrazolium bromide (MTT) technique. Briefly, the compounds were applied to cell cultures at defined concentration intervals (1.65 µg/mL to 100 µg/mL) for 24 h. At the end of the incubation period, 10 µL of the MTT solution (5 mg/L) was added to each culture well, and the samples were incubated at 37 °C for 3 h. Then, the culture mediums were discarded, and 100 µL of DMSO was added to the samples to dissolve formazan crystal to obtain a purple/blue color. A microplate reader was used to measure the color intensities at a 570 nm wavelength.

### 2.3. Hoechst 33258 Fluorescent Staining and Nuclear Abnormality Analysis

The Hoechst 33258 nuclear staining method was used to monitor abnormalities in the nuclear structures of a healthy HDFa cell culture. Tested drugs were applied to the HDFa cell cultures at various concentrations, and the cell cultures were incubated at 37 °C for 24 h. Then, HDFa cells were fixed by using 4% paraformaldehyde at 4 °C for 30 min. After the incubation period, the cell cultures were washed with PBS two times. A total of 1 µM Hoechst 33258 fluorescent dye was added to the samples and incubated at room temperature for 5 min. Nuclear morphologies were monitored and photographed under a fluorescent microscope (Leica^®^ DM IL LED, Wetzlar, Germany).

### 2.4. Flow Cytometry Analysis

Flow cytometry analysis was performed by using the FITC Annexin V/Dead Cell Apoptosis Kit with FITC Annexin V and PI according to the producer’s recommendations. Further, 5 × 10^4^ cells were collected by trypsinization and centrifugation. The cells were resuspended in 500 μL of 1× binding buffer and 5 μL of propidium iodide, and the Annexin V-FITC (50 μg/mL) dyes were added to the cultures and incubated in the dark for 5 min. Finally, the cultures were analyzed via the use of flow cytometry (The CyFlow^®^ Cube 6, Norderstedt, Germany).

### 2.5. Statistical Analyses

The GraphPad Prism (GraphPad Software^®^, Version 8, San Diego, CA, USA) statistical program was used to calculate significant differences between experimental groups. Analyses were performed via the use of one-way ANOVA and the Dunnett comparison test. The statistically significance level was accepted for each group as *p* < 0.05.

### 2.6. Molecular Docking Analysis

**Figure 1:**
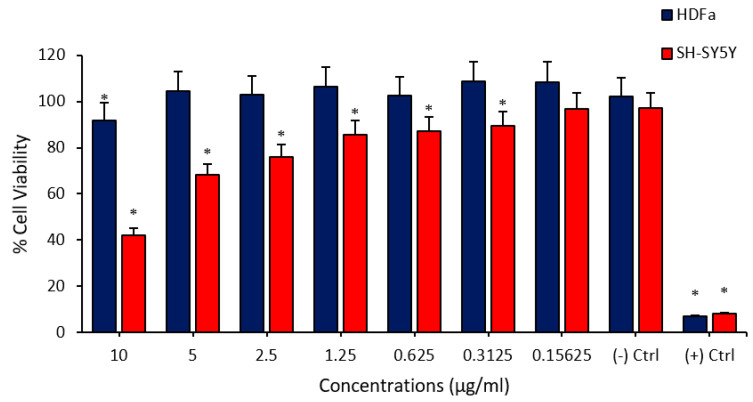
Determination of the cytotoxic effects of PTX applied at different concentrations on human fibroblast (HDFa) cells and SHSY-5Y neuroblastoma cells by MTT viability test. The asterisk (*) symbol indicates a significant decrease in viability on cells (*p* < 0.05).

Docking simulations were conducted by employing AutoDock software (version 4.2.6), sourced from the official website of the Scripps Research Institute (http://autodock.scripps.edu/, accessed on 12 November 2023). The simulations utilized Python 3.10.5 and MGLTools 1.5.7. AutoDock employed a genetic algorithm (GA) during the optimization phase, executing 10 runs in the GA processes, following a previously established protocol [18]. The crystal structure of Human Complexed with Troglitazone (2VN0) was retrieved from the Protein Data Bank (RCSB PDB, https://www.rcsb.org/, accessed on 11 January 2023). The molecule structure for favipiravir was obtained from the National Library of Medicine (PubChem, https://pubchem.ncbi.nlm.nih.gov/, accessed on 11 January 2023). In the initial stages of the docking process, water molecules were eliminated and hydrogen atoms were added to prepare the protein using AutoDock software. Docking data from AutoDock were analyzed using the online Protein–Ligand Interaction Profiler (plip-tool.biotec.tu-dresden.de, accessed on 18 November 2023). Detailed calculations of the distances between ligands and amino acid residues were performed. Visualization of the 3D residual interaction plots was accomplished using PyMol software (pymol.org, accessed on 18 November 2023). Additionally, LigPlot software (Version 4.5.3, https://www.ebi.ac.uk/thornton-srv/software/LIGPLOT, accessed on 18 January 2023) was employed to generate 2D sketches, providing insights into the interactions between the protein and ligand [19,20]. Finally, a comparative analysis between the theoretical data obtained from AutoDock software and the experimental results was presented. Strikingly, a high level of agreement was observed between these results. Figure 1 illustrates the visualization of binding affinities, hydrogen bonds and other interactions between the protein and ligand, as determined by AutoDock 4.2.6.

## 3. Results

**Figure 2:**
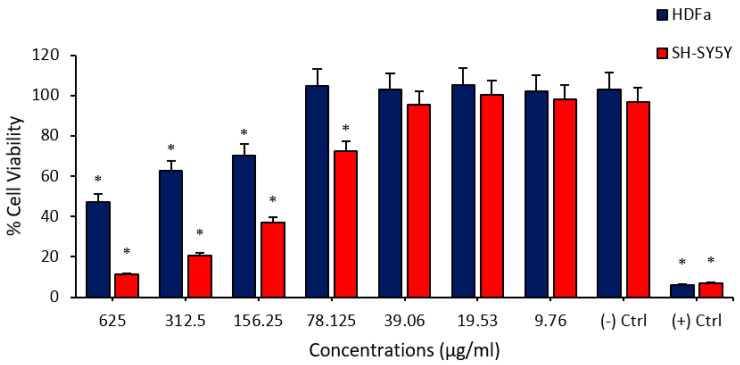
Determination of the cytotoxic effects of FPV administered at different concentrations on human fibroblast (HDFa) cells and SHSY-5Y neuroblastoma cells by MTT viability assay. The asterisk (*) symbol indicates a significant decrease in viability on cells (*p* < 0.05).

In the first part of the study, biosafety and anticarcinogenic analyses of FPV and PTX were performed by using cytotoxicity analyses. It was observed that PTX administration to HDFa and SHSY-5Y cells at different concentration ranges (0.15625–10 µg/mL) caused remarkable cytotoxicity at certain concentrations. It was determined that PTX showed a higher cytotoxic property in the neuroblastoma cell line and caused a significant decrease in cell viability, even at concentrations of 0.3125 µg/mL. In addition, it was understood that the PTX application to healthy cells has a significant cytotoxic effect only at a concentration of 10 µg/mL and did not cause any change in cell viability at lower doses (Figure 1). Cell viability studies performed on FPV showed significant cytotoxicity in HDFa and SHSY-5Y cell lines at high concentrations. According to these analyses, FPV administered at concentrations between 625 µg/mL and 78,125 µg/mL exhibited a cytotoxic effect on the SHSY-5Y cell line. In addition, FPV applied at concentrations of 625 µg/mL, 312.5 µg/mL and 156.25 µg/mL caused a viability-reducing effect on HDFa cell cultures (Figure 2).

**Figure 3:**
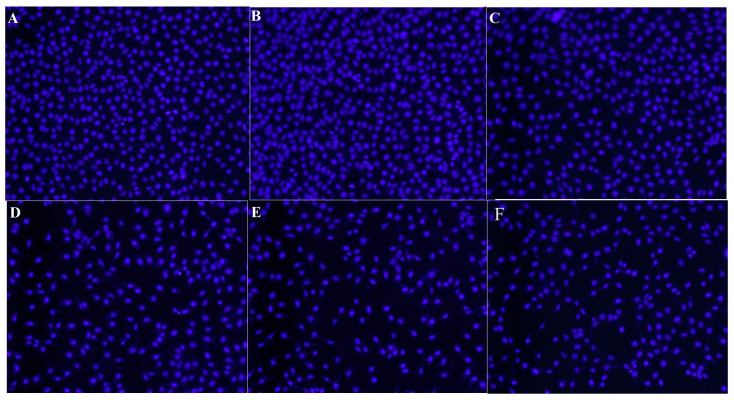
Nuclear structures of HDFa healthy cell cultures treated with FPV and PTX by Hoechst 33258 fluorescent core staining method. (**A**) Negative control, (**B**) FPV (9.7 µg/mL), (**C**) FPV (19.5 µg/mL), (**D**) FPV (39 µg/mL), (**E**) PTX (10 µg/mL) and (**F**) FVP (39 µg/mL) + PTX (10 µg/mL).

The genotoxic effects that might occur in HDFa healthy cells with Hoechst 33258 fluorescent nuclear staining analyses were performed within the scope of biosafety analyses and monitored under an inverted fluorescent microscope to observe micronucleus (MN) structures. In line with the analyses performed, no significant numerical anomaly change was observed in the HDFa healthy cell line treated with FPV and PTX as compared to the negative control (HDFa cell line with no treatment) (Figure 3 and Table 1).

In order to determine the synergistic effects of FPV and PTX, the effects of the MTT cell viability analyses were determined by co-administration of these two compounds in the SHSY-5Y cancer line. In the study, the highest toxic dose of 10 µg/mL was applied for PTX. This application was carried out by applying PTX and FPV alone together with three non-toxic doses (fd1: 39.06 µg/mL, fd2: 19.53 µg/mL and fd3: 9.76 µg/mL) to the SHSY-5Y cell culture. According to the results, it was observed that PTX at a concentration of 10 µg/mL applied to the SHSY-5Y cell culture alone caused a 37% viability on the cell culture. It was determined that all non-toxic FPV concentrations administered with PTX reduced the viability values to a lower level (Supplementary Figure S1).

**Figure 4:**
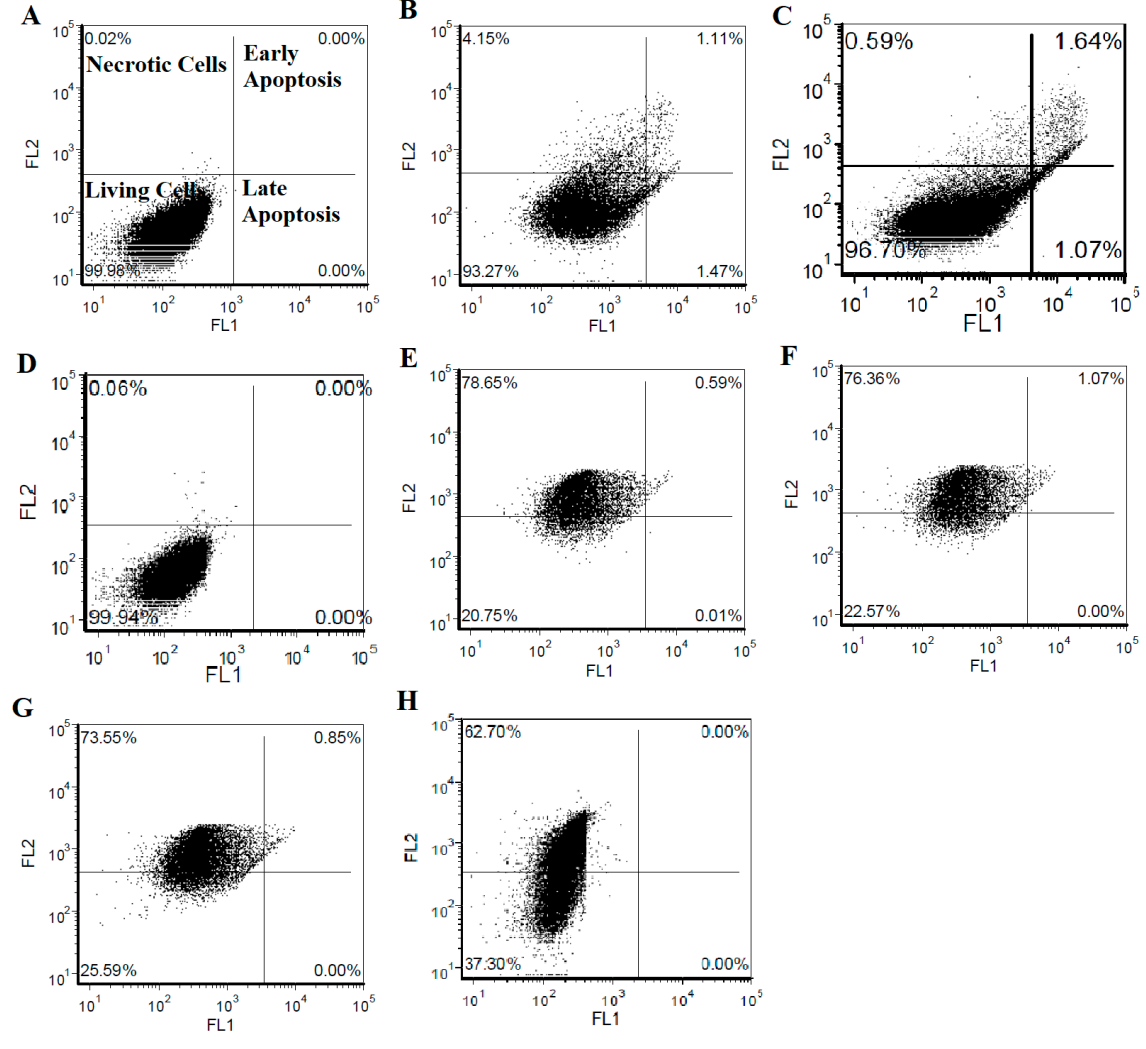
Determination of the synergistic effect of FPV and PTX on SHSY-5Y neuroblastoma cells by flow cytometry analysis. (**A**) Negative control, (**B**) only FPV application at 39.06 µg/mL concentration, (**C**) only FPV application at 19.53 µg/mL concentration, (**D**) only FPV application at 9.76 µg/mL concentration, (**E**) 39.06 µg /mL of FPV and 10 µg/mL of PTX application, (**F**) 19.53 µg/mL of FPV and 10 µg/mL of PTX application, (**G**) 9.76 µg/mL concentration FPV and 10 µg/mL concentration PTX application and (**H**) only 10 µg/mL of PTX application.

In addition, the synergistic effect of PTX and FPV on the SHSY-5Y cell culture was confirmed by flow cytometry analysis. In line with the analyses performed, it was shown by flow cytometry that the applied FPV concentrations did not cause a significant change in cell viability (Figure 4B–D). On the other hand, it is understood that a 10 µg/mL PTX application causes about 37% viability in cell cultures (Figure 4H). However, co-administration of PTX with non-toxic FPV concentrations produced a synergistic effect, further reducing cell viability under a 37% ratio (Figure 4E–G).

**Figure 5:**
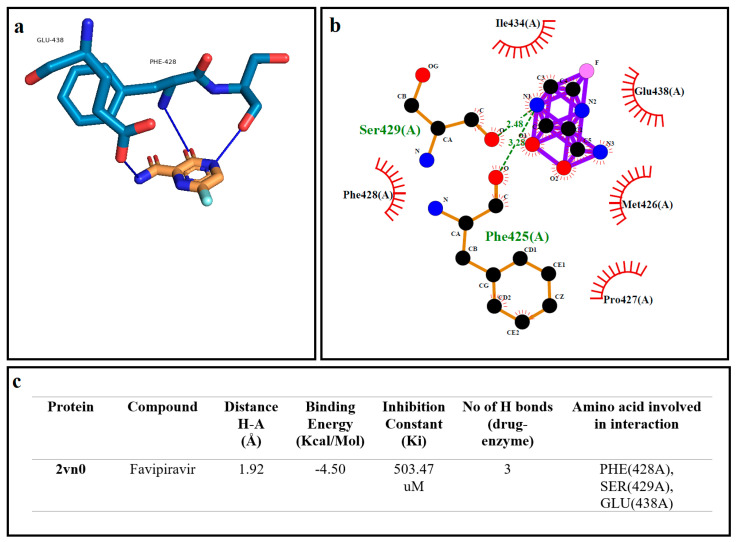
The best docking formation of the binding mode of CYP2C8DH complex (2VN0) with Favipiravir obtained by (**a**)—PyMol, (**b**)—LigPlot, and (**c**)—the table of hydrogen bonds (H–bonds) (with distance Å), binding energy (Kcal/mol), inhibition constant, number of H–bonds (drug–enzyme) and amino acid involved in interaction obtained from the Protein–Ligand Interaction Profiler. The blue line and dashed–gray show the H–bonds and hydrophobic interactions in PyMol, respectively.

In the molecular docking study, the binding affinity and interaction profile of the CYP2CDH enzyme with Favipiravir were elucidated. PyMol and LigPlot visualizations revealed an optimal binding mode with Favipiravir snugly positioned within the active site of CYP2CDH, characterized by a binding energy of −4.50 Kcal/mol and an inhibition constant of 503.47 µM (Figure 1). The docking formation displayed three hydrogen bonds with the amino acids SER (428A), PHE (429A) and GLU (438A), with the bond distances suggesting favorable interactions, particularly a hydrogen bond with a distance of 1.92 Å. The interaction profile, corroborated by the Protein–Ligand Interaction Profiler, highlighted a network of hydrophobic and hydrogen bond interactions, where the blue lines and dashed-gray lines in the PyMol visualization represented these interactions, respectively. This detailed interaction map contributes to our understanding of the molecular mechanisms by which Favipiravir may exert its inhibitory effects on the CYP2CDH enzyme (Figure 5).

## 4. Discussion

The global COVID-19 pandemic has driven researchers to develop drugs or vaccines to prevent or stop the progression of this disease. Also, previously utilized antiviral drugs are being evaluated to speed up the treatment process [4,21,22]. FPV is a drug approved for the pandemic flu that originated in Japan and is stated to be effective against severe acute respiratory syndrome coronavirus-2. It has a wide margin of therapeutic safety for high-dose uses and has demonstrated rapid viral clearance and a superior recovery rate in clinical trials of COVID-19 [23,24]. Generally, studies on FPV showed promising results in clinical trials in many countries, including the UK, US and India. Also, positive results in the treatment of COVID-19 were shown in many other countries that prove the effectiveness of FPV [25,26].

The focus on FPV as a CYP2C8 inhibitor in research, among the plethora of drugs known to inhibit this enzyme, is influenced by several key factors. Firstly, FPV’s specificity and interaction profile with CYP2C8 may be distinct, offering unique insights into drug metabolism and interactions. In fact, the concentration-dependent inhibitory effect on CYP2C8 by FPV was reported with an IC_50_ value of 74.9 μg/mL [27]. Secondly, the clinical relevance of FPV, particularly in the treatment of COVID-19, underscores the importance of understanding its pharmacokinetic properties in the context of widespread use and polypharmacy, especially in patients with comorbid conditions [28]. FPV is a prodrug with high bioavailability and moderate protein binding. It has a limited distribution in the body, and its peak concentration in blood occurs relatively quickly after administration. The drug is eliminated rapidly through the kidneys, and its elimination process is influenced by both dosage and time. FPV does not undergo metabolism via the cytochrome P450 system but can inhibit one of its components, making caution necessary when used with other drugs processed by this system. Additionally, investigating FPV’s role as a CYP2C8 inhibitor can enhance our understanding of potential drug–drug interactions, which is crucial for patient safety and effective medication management. The recent emergence and widespread availability of FPV for COVID-19 treatment further motivate the need to study its interactions with other drugs metabolized by CYP2C8. This research is not only significant for optimizing therapeutic strategies but also for mitigating the risk of adverse drug reactions, particularly in the current landscape of COVID-19 treatment, where polypharmacy is common.

The pivotal role of cytochrome P450 (CYP) 2C8 in drug metabolism has been increasingly recognized over the last decade. This enzyme, predominantly located in the liver and also present in the brain, is integral to the metabolic processing of a diverse range of pharmaceuticals. The spectrum of drugs influenced by CYP2C8 is extensive, encompassing various classes, such as statins, antimalarials, anticancer agents and antidiabetics. Key drugs metabolized by CYP2C8 include cerivastatin, amodiaquine and paclitaxel, among others. The expanding list of drugs identified as substrates for CYP2C8 in the recent literature underscores the enzyme’s significant role in determining drug efficacy and safety. This growing understanding of CYP2C’s role in drug metabolism is crucial for optimizing drug therapy and personalizing medical treatments. [29]. The metabolism of PTX by CYP2C8 is significant and has been explored in various studies. Cytochrome P450 (CYP) 2C8 is identified as the principal enzyme responsible for PTX metabolism. This role is critical, as polymorphisms in CYP2C8 can lead to variations in PTX metabolism, impacting drug efficacy and safety. For instance, certain genetic variants in CYP2C8 are associated with decreased PTX metabolism, which can influence the pharmacokinetics and, potentially, the therapeutic outcomes of PTX treatment. These findings highlight the importance of considering CYP2C8 activity and genetic variations when administering PTX, especially in personalized medicine approaches where individual genetic profiles are considered for optimizing drug therapy [30,31].

Many drugs have been studied as inhibitors or inducers of CYP2C8, and recent studies showed that the acyl-β-glucuronides of clopidogrel and gemfibrozil cause metabolism-dependent inactivation of CYP2C8, resulting in a strong drug activity enhancement in cellular models. Furthermore, various glucuronide metabolites cooperate with CYP2C8 as substrates or inhibitors, showing a strong interaction between CYP2C8 and glucuronides [32,33,34]. FPV was shown to be not a CYP450 substrate but was claimed to be a possible CYP2C8 inhibitor [35]. Therefore, co-administration of FPV with CYP2C8 substrates could raise the risk for enhanced serum levels of xenobiotics and result in the toxic effects of excessive drug administration. FPV was also shown to inhibit acetaminophen metabolism, which could increase the toxicity risk at higher doses for long periods. Moreover, it was claimed that drug co-administrations, such as tapentadol and tramadol with FPV, may increase acetaminophen toxicity [36,37,38].

In the present study, we aimed to determine the ability of the use of FPV to create a toxic state in cancer treatment with PTX through synergistic effect studies. Biosafety analyses of FPV and PTX, which were used in the direction of our study, were performed on the HDFa healthy cell line, and it was investigated whether these compounds already have cytotoxic and genotoxic effects on healthy tissue. The use of healthy fibroblast cell lines in brain cancer drug research is important for understanding drug effects on non-cancerous cells, which is critical for assessing side effects and systemic toxicity. This approach is supported by the National Cancer Institute, which emphasizes the need to evaluate both the therapeutic and toxic effects of anticancer agents on various cell types, including fibroblasts. Since fibroblasts share important cellular processes with neuronal cells, such as DNA repair and cell-cycle regulation, studying them helps us gain insights into the potential impacts of drugs on neuronal cells. Moreover, their stability and ease of culturing make fibroblasts a practical choice for initial drug screening in cancer research [39,40]. According to our results, it was determined that the applied agents caused cytotoxicity in healthy cells at certain dose ranges and did not show a significant genotoxic feature in comparison with the control group. Based on our subsequent anticarcinogenicity analyses, it was confirmed that PTX has a high cytotoxic effect in the SHSY-5Y cancer line, even at low concentrations. In order to determine the synergistic properties, non-cytotoxic doses of FPV were also evaluated on the SHSY-5Y cancer cell line. As a result of applying these two agents together in the cancer cell culture, it was shown by both cell viability analysis and the flow cytometry technique that FPV applied at non-toxic concentrations significantly increased the cytotoxicity of PTX.

## 5. Conclusions

In light of the present findings, it is revealed that FPV, which is used in the treatment of COVID-19 under normal conditions, does not have a genotoxic or highly cytotoxic effect, but it is thought that it can play a potential enzyme inhibitor role. At the same time, we suggest that PTX, which is frequently used in cancer treatment, has the potential to produce increased cytotoxicity, which could cause systemic toxicity if administered with FPV to a patient receiving COVID-19 treatment. At the same time, a validation of the results by conducting studies such as enzyme activity, organ toxicity and drug interactions will enable a reconsideration of drug dose adjustments and determination of more appropriate dose amounts in cancer patients treated with COVID-19.

This study stands out for its innovative approach, particularly in examining the synergistic effects of FPV and PTX in the context of neuroblastoma cells, which is highly relevant given the ongoing COVID-19 pandemic and its implications for cancer patients. The utilization of advanced techniques, such as fluorescent microscopy and flow cytometry, adds a layer of scientific rigor and provides comprehensive insights into the cellular mechanisms at play. Furthermore, the potential clinical relevance of these findings cannot be overstated, as the study suggests that the anticancer properties of PTX could be enhanced when used in conjunction with FPV, a finding that could have significant implications for cancer treatment, especially for patients concurrently receiving antiviral treatment. On the other hand, the study presents several inherent limitations. Being an in vitro study, it is crucial to acknowledge that these findings might not fully translate to in vivo conditions or human physiology. The human body’s complexity and internal interactions cannot be fully replicated in a laboratory setting. Moreover, the absence of long-term data in the study is a significant gap, as understanding the chronic side effects or long-term benefits of combined FPV and PTX treatment is crucial for clinical application.

## Figures and Tables

**Table 1:** The rates of micronuclei (MN), lobed (L) and notched (Ntc) nuclei anomalies in HDFa healthy cell cultures treated with PTX and FPV according to Hoechst 33258 fluorescent nuclei staining analysis. One-way ANOVA and Dunnett tests were used for multiple comparisons.

Compounds	MN	L	Ntc	Abnormality Numbers/1000 Cells
Negative Control	7	4	6	0.017 ± 0.013 a
FPV (9.7 µg/mL)	8	7	5	0.020 ± 0.008 a
FPV (19.5 µg/mL)	6	6	7	0.019 ± 0.016 a
FPV (39 µg/mL)	7	7	5	0.019 ± 0.007 a
PTX (10 µg/mL)	8	5	6	0.019 ± 0.013 a
FPV (9.7 µg/mL) plus PTX (10 µg/mL)	7	6	7	0.020 ± 0.011 a
FPV (19.5 µg/mL) plus PTX (10 µg/mL)	8	8	4	0.020 ± 0.014 a
FPV (39 µg/mL) plus PTX (10 µg/mL)	6	5	8	0.019 ± 0.014 a

^a^: shows statistically significant results compared to each other.

## Data Availability

The authors confirm that the data supporting the findings of this study are available within the article and its Supplementary Material.

## Supplementary Materials

The following supporting information can be downloaded at: https://www.mdpi.com/article/10.3390/medicina60010082/s1, Figure S1: Determination of the cytotoxic effects of FVP plus PTX applied at different concentrations on human fibroblast (HDFa) cells and SHSY-5Y neuroblastoma cells by MTT viability test.
