# Supplementary material for: Drug Synergism of Anticancer Action in Combination with Favipiravir and Paclitaxel on Neuroblastoma Cells
Source: Medicina (Kaunas). 2023 Dec 30;60(1):82. doi: 10.3390/medicina60010082 (PMC10820816; doi:10.3390/medicina60010082)
Supplement: Supplementary file 1 [file medicina-60-00082-s001.zip › medicina-2752562-supplementary.pdf]

Supplementary Data

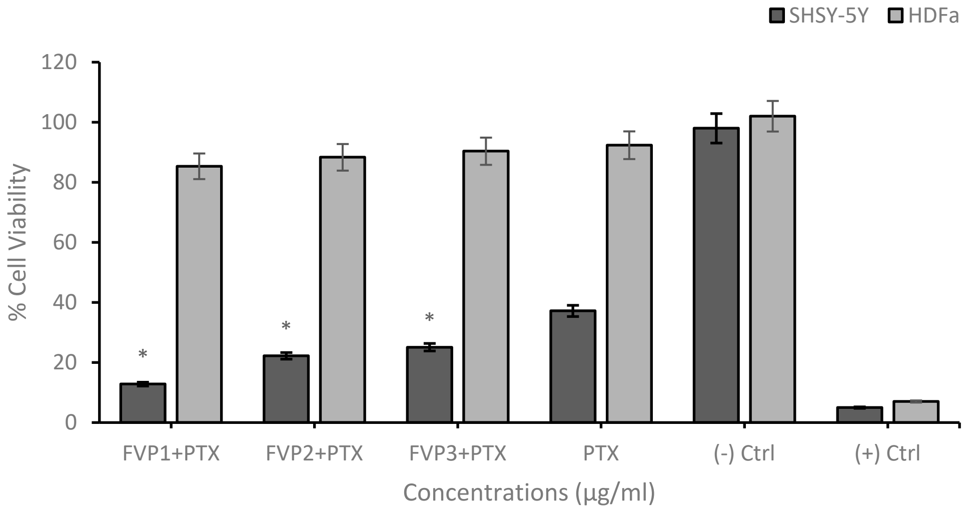

Supplementary Figure S1. Determination of the cytotoxic effects of FVP plus PTX applied at different concentrations on human fibroblast (HDFa) cells and SHSY-5Y neuroblastoma cells by MTT viability test. (FVP1: 39.06 µg/ml, FVP2: 19.53 µg/ml, FVP3: 9.76 µg/ml and PTX: 10 µg/ml) The asterisk (*) symbol indicates a significant difference in viability on cells compared to PTX only group (P<0.05).
